# Take Warning!

**Published:** 1907-07

**Authors:** Gustavus North

**Affiliations:** Cedar Rapids, Iowa.


					TAKE WARNING!
By Dr. Gttstavtts North, Cedar Rapids, Iowa.
I notice the following article in the May number of ' Hints
Department" of the American Journal of Dental Science
copied from the Western Dental Journal.
"When and When not to Extract.?(a) If not more than
three scattered teeth remain extract them, (b) If two
molars or bicuspids remain on each side, do not extract,
(c) If only the incisors remain, extract, (d) If four or five
teeth remain on one side of the jaw and none on the opposite
side extract."
I do not know who is the author of this short article. If
it is the editor of the Western Dental Journal I am sur-
prised, and if it is not I am surprised to think he would pub-
lish such an article in his journal to be read and absorbed by
the profession, without editorial condemnation.
It brought tears to my eyes when I read the article, to
think any one would advocate such practice in this day and
age of the world.
170 THE AMERICAN JOURNAL
Dentists are a progressive class of professional men, and
are not going back, I hope to primitive ideas. I seldom ex-
tract teeth, and then only when they are beyond saving. If
I only find one or two firm teeth in the mouth, I save them.
I often crown roots, and then make partial dentures.
I admit it is easier for dentists to make a clean sweep in
extracting and making artificial dentures, but the dentist
should not take this into consideration.
God bless the dentist that saves teeth instead of destroy-
ing them, is my prayer.

				

